# Anthelmintic resistance in sheep nematodes in Australia: a compilation of recent test results

**DOI:** 10.1111/avj.70081

**Published:** 2026-04-11

**Authors:** RB Besier, NM Rolls

**Affiliations:** ^1^ Brown Besier Parasitology Pty Ltd Albany Western Australia 6330 Australia; ^2^ School of Agricultural Science Murdoch University Murdoch Western Australia 6150 Australia; ^3^ Elanco Australasia Pty Ltd 3/7 Eden Park Drive Macquarie Park New South Wales 2113 Australia

**Keywords:** anthelmintic resistance, faecal egg count reduction test, sheep nematodes

## Abstract

**Objective:**

The results of recent faecal egg count reduction tests (FECRTs) were collated to provide an indication of the prevalence and severity of anthelmintic resistance in sheep nematodes across Australia and of changes in comparison to earlier testing reports.

**Methods:**

FECRTs conducted in New South Wales, South Australia, Western Australia and Victoria from 2021 to 2024 were analysed to indicate relative efficacy against the nematodes *Haemonchus contortus*, *Teladorsagia circumcincta* and *Trichostrongylus* species. The prevalence and percentage reductions were compared to previous reports of anthelmintic resistance in Australia.

**Results:**

The efficacy of a variety of anthelmintics was assessed in FECRTs in several Australian states, with up to 80 tests for the broad‐spectrum anthelmintics and at least 20 tests for closantel and naphthalophos. Resistance (a reduction in worm egg counts of less than 95%) in any one of the major nematodes *Haemonchus contortus, T. circumcincta* or *Trichostrongylus* species was seen for albendazole, levamisole, abamectin and moxidectin in 96%, 88%, 91% and 84% of tests, respectively, and in 66% of tests for a benzimidazole–levamisole–abamectin combination. For the newer anthelmintic groups, the comparable prevalence was 23% for both monepantel and a derquantel–abamectin combination, but the comparative efficacy (reduction in worm egg count) was considerably greater than for the older anthelmintics. For individual worm species, resistance in *T. circumcincta* was common and often severe to the benzimidazoles, levamisole and macrocyclic lactones; in *H. contortus* to the benzimidazoles and macrocyclic lactones; and in *Trichostrongylus* mostly to the benzimidazoles and levamisole. For *H. contortus*, closantel resistance was seen in 60% of tests, but resistance in this species was less prevalent for levamisole (21% of tests) and naphthalophos (31% of tests). For all species and anthelmintics, resistance had increased in comparison to previously reported figures.

**Conclusion:**

The FECRT figures suggest that there is little role for the benzimidazole, levamisole and macrocyclic lactone groups as single actives and that all anthelmintics will be used most effectively and sustainably as combinations of actives, but their efficacy on individual sheep properties should be confirmed with an FECRT.

**Abbreviations**
epg: eggs per gram
FECRT: faecal egg count reduction test
FWEC: faecal worm egg count
IPM: integrated parasite management
ML: macrocyclic lactone

The effective control of sheep nematodes is essential for efficient sheep production, as the mortalities and production loss related to poorly controlled worm infections are a significant restriction on profitability. In Australia alone, the combined effect of nematode infections has been estimated to cost the sheep industries some A$665 million annually, of which the great majority ($562 million) is production loss.^1^ Sheep worm control continues to rely largely on the use of anthelmintics and any reduction in their effectiveness decreases the efficiency of control measures, but effective control is compromised by anthelmintic resistance, which has been reported from most countries with significant sheep populations.^2^, ^3^

In Australia, anthelmintic resistance (generally defined as less than a 95% reduction in faecal worm egg count in comparison to an untreated control group of sheep^4^) was first recognised in *Haemonchus contortus* in the late 1960s and subsequently in other important nematodes, including *Teladorsagia circumcincta* and various *Trichostrongylus* species.^5^ Resistance had become widespread to ivermectin, the first of the macrocyclic lactone (ML) anthelmintics for sheep, within 10 years of its introduction to Australia, and multiple resistance (affecting several worm genera and different anthelmintics) had also been detected.^4^ More recently, the results of a large number of tests on properties throughout Australia over a 4‐year period to 2012 indicated resistance to at least one anthelmintic in at least one of the genera *Haemonchus*, *Teladorsagia* or *Trichostrongylus* on almost all properties tested.^6^ Resistance was detected commonly to all MLs, including abamectin and moxidectin, and in some 28% of groups treated with a ‘triple combination’ of a benzimidazole, levamisole and an ML. Similarly, a collation of results from 66 drench tests from the state of Victoria over the period 2012 to 2018 indicated resistance to be common for the MLs and also present for some ‘triple combination’ (benzimidazole, levamisole and an ML) products.^7^ Resistance to the more recently introduced anthelmintics was not reported in either recent compilation but has been confirmed in sheep in Australia for both monepantel^8^, ^9^ and derquantel in combination with abamectin.^10^, ^11^

The present report of faecal egg count resistance testing (FECRT) results provides a more recent indication of the extent and severity of anthelmintic resistance, with results from 80 tests conducted across Australia in the years 2021 to 2024.

## Materials and methods

### Resistance test procedures

The drench resistance tests were conducted on properties in four states of Australia (Figure 1) which were identified by representatives of the animal health company Elanco, on the basis of a large flock size (typically 5000 breeding ewes or more) and stated interest by sheep owners in effective worm control (including an informed approach to anthelmintic selection). The tests were conducted using young sheep (mainly current‐year lambs) before they had received any anthelmintic treatment, or otherwise where no anthelmintic had been administered within the previous 6 weeks, and during the main worm risk season for each location. A pre‐test faecal sampling was conducted on candidate flocks to ensure an adequate worm count and that the nematode species typical of the environment were present.

**Figure 1:**
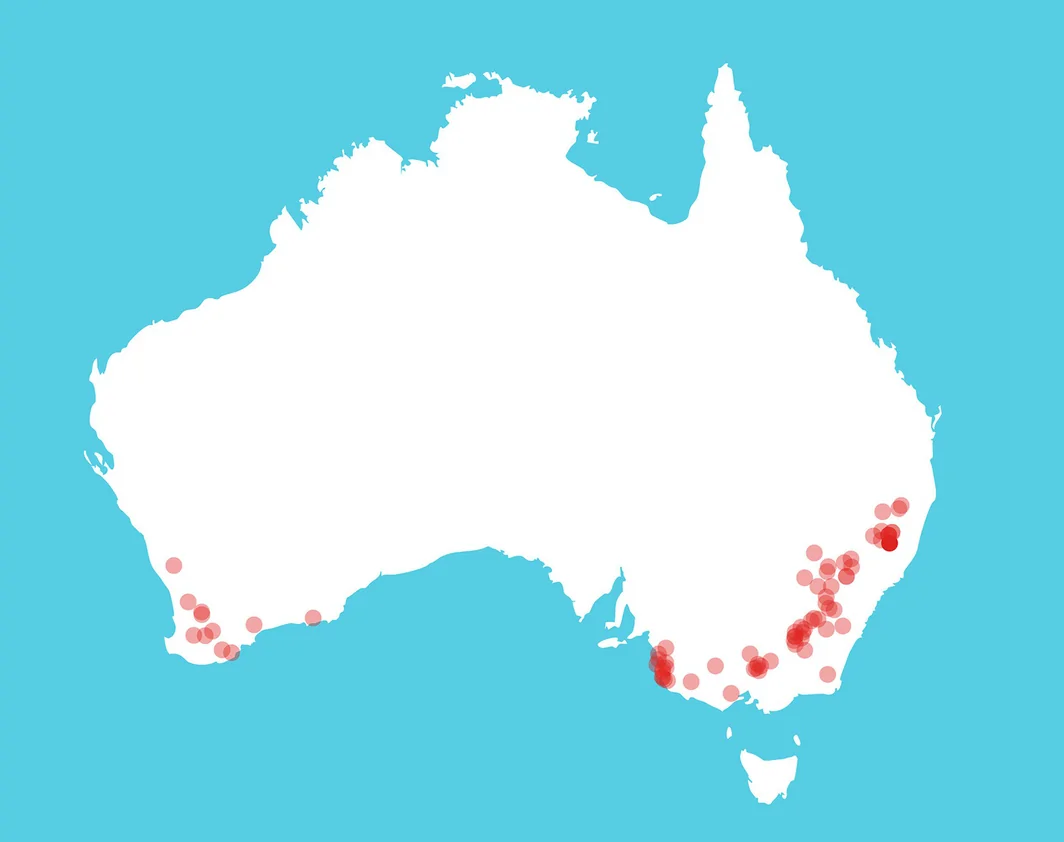
Locations of properties of anthelmintic resistance tests.

The faecal worm egg reduction test protocol followed the recommendations of the 1995 WAAVP guideline^12^ and the Wormboss website (https://wormboss.com.au/tests-tools/testing-drench-effectiveness-with-a-drenchtest/). Sheep were randomly assigned to treatment groups of 12 for each anthelmintic tested, plus an untreated Control group, and identified to group by separately coloured ear tags for each. The sheep were weighed on Day 1 of the test, and drench doses administered to the weight of individual sheep using a separate syringe for each group. Between 10 and 14 days after treatments, sheep were yarded and faecal samples taken from the first 10 in each group from which faeces could be collected. Samples from individual sheep were kept separate after collection and forwarded to the laboratory to arrive within the minimum time (before worm egg development was evident).

The panel of drenches aimed to encompass the major anthelmintic groups or commonly used products and included albendazole, levamisole, the MLs abamectin and moxidectin, a derquantel–abamectin combination (‘Startect’), monepantel (‘Zolvix’) and an abamectin–benzimidazole–levamisole combination, with some variation between properties of the actual brand names. In regions where *H. contortus* is especially prevalent, closantel was included in most tests, and naphthalophos (commonly used against several species, usually in combination with other anthelmintics) was also included.

### Laboratory procedures

The laboratory procedures followed were standard for anthelmintic resistance testing in Australia,^13^ with faecal worm egg counts (FWECs) performed using a modified McMaster method, with 2.5 g per sample and a sensitivity of one egg detected in counting chambers representing 60 eggs in the initial sample. Larval cultures were prepared with approximately equal quantities of faecal material from each sheep, incubated for 7 days at 26°C–28°C, and larval differentiations conducted using standard identification keys.^14^, ^15^

### FWEC reduction calculations

Reductions in FWEC were conducted using the statistical program ‘RESO’, an anthelmintic resistance calculator running on an Excel spreadsheet (Microsoft Corporation, USA), initially developed by L. Wursthorn and P. Martin (CSIRO, 1990) and more recently modified by Robert Dobson (Murdoch University). Tests were included in the database only if considered to provide adequate sensitivity, that is, where the minimum all‐species mean Control group FWEC was at least 150 eggs per gram (epg), or for individual genus/species calculations, a minimum of 50 epg for a single genus/species, provided that the larval differentiation figure for a group was a minimum of 5%. Where only a single egg was detected in a single sheep within a test group, this was ignored to account for the possible cross‐contamination from another group, and the group considered to have a zero count.

The percentage reduction was calculated initially for all worm eggs (i.e. undifferentiated for genus/species identity) and then individually for the nematodes *H. contortus*, *T. circumcincta* and *Trichostrongylus* species (the latter differentiated to genus only), as:

Reduction % = 100 × (1 − [mean count of the treated group]/[mean count of the control group]), based on arithmetic group means. ‘Anthelmintic resistance’ was defined as a FWEC reduction of less than 95% in comparison to the control count of a particular species or genus, and a minimum lower confidence interval of less than 90%.

## Results

A total of 80 tests providing acceptable sensitivity for at least one of the major nematode species *Haemonchus contortus*, *T. circumcincta* or ovine *Trichostrongylus* species were conducted during the calendar years 2021, 2022, 2023 and 2024 (13, 29, 27 and 11 tests, respectively), in the states of New South Wales (47 tests), South Australia (12 tests), Western Australia (11 tests) and Victoria (10 tests). The mean all‐property FWEC for the untreated Control group was 1894 epg, with overall larval differentiation results of 44% for *H. contortus*, 32% for *Trichostrongylus* and 20% for *T. circumcincta* (and approximately 4% of other species). The mean all‐property count for the Control group was 1623 epg for *H. contortus*, 489 epg for *Trichostrongylus* and 227 epg for *T. circumcincta*, with maximum counts of 9259 epg, 3841 epg and 1792 epg, respectively.

Reductions of less than 95% in faecal worm egg count to any one of the three major species were indicated to at least one species on the great majority of the properties where the older anthelmintic groups were tested (Table 1, ‘Any Species’): in all except 3 of 80 tests for albendazole (i.e. 96% of tests) and in 88%, 91% and 84% of tests for levamisole, abamectin and moxidectin, respectively. In comparison, for the newer anthelmintics, resistance to one or more species was found in 23% of tests each for derquantel–abamectin and monepantel and in 66% of 44 tests for the ‘triple combination’ of a benzimidazole, levamisole and abamectin. A smaller number of tests were conducted for the narrow‐spectrum anthelmintics, with less than 95% reduction in 60% of 25 tests for closantel (specifically for *H. contortus*) and 62% of 21 tests for naphthalophos (for any species). The worm egg count reductions for nematodes other than *H. contortus, T. circumcincta* or *Trichostrongylus* included the genera *Oesophagostomum, Chabertia* and *Cooperia*, but no reductions of less than 95% were recorded for any of these in any test. Including these nematodes in the total of undifferentiated counts resulted in little change in overall percentage reductions, and none in the inferences regarding the prevalence or severity of resistance.

**Table 1:** Resistance prevalence: number and percentage of FECR tests where worm egg counts were reduced by <95% for each of the differentiated species/genus *H. contortus, T. circumcincta* or *Trichostrongylus*, or for any of these species in a single test

Anthelmintic or group	*H. contortus* % tests (n/n)	*T. circumcincta* % tests (n/n)	*Trichostrongylus* spp % tests (n/n)	Any species^a^ % tests (n/n)
Albendazole	80 (45/56)	94 (50/53)	84 (54/64)	96 (77/80)
Levamisole	21 (12/56)	94 (50/53)	67 (43/64	88 (65/80)
Abamectin	95 (53/56)	83 (44/53)	30 (19/64)	91 (69/80)
Moxidectin	79 (44/56)	70 (37/53)	30 (20/64)	84 (58/80)
Benzimidazole/levamisole/abamectin combination	7 (2/27)	67 (24/36)	28 (12/40)	66 (29/44)
Derquantel–abamectin combination	13 (7/56)	21 (11/53)	3 (2/64)	23 (18/80)
Monepantel	13 (7/55)	15 (8/53)	4 (4/64)	23 (18/79)
Naphthalophos	33 (6/18)	*	82 (9/11)	62 (13/21)
Closantel	60 (15/25)	**	**	**

^a^ Any of the nematodes *H. contortus, T. circumcincta* or *Trichostrongylus* species (less than 95% reduction).

* Too few tests for useful figures.

** Closantel has no significant activity against species other than *H. contortu*s.

The prevalence of resistance in different nematodes (Table 1) was greatest for albendazole and indicated in 94%, 84% and 80% of tests for *T. circumcincta*, *Trichostrongylus* spp and *H. contortus*, respectively, with mean reduction percentages (efficacy or severity; Table 2) of 35%, 67% and 65%, respectively. However, resistance varied between genera for the other anthelmintic groups, with levamisole resistance common and severe in *T. circumcincta* (94% of tests, with a mean FWEC reduction of 49%) but uncommon in *H. contortus* (21% of tests; mean FWEC reduction of 92%). In contrast, for the MLs, resistance was particularly severe in *H. contortus*, with a prevalence for abamectin resistance of 95% and a mean FWEC reduction of only 26%, although slightly less pronounced for the more potent ML, moxidectin (prevalence of 79% of tests and a mean efficacy of 67%). However, resistance to this class was much less severe in *Trichostrongylus*, with a prevalence of 30% each for both abamectin and moxidectin, and mean FWEC reductions of 89% and 92%, respectively.

**Table 2:** Resistance severity: mean efficacy (percentage reductions of worm egg counts) for each of the differentiated species/genus *H. contortus, T. circumcincta* or *Trichostrongylus* ^a^

Anthelmintic or group	*H. contortus* (N tests)	*T. circumcincta* % tests (n/n)	*Trichostrongylus* spp % tests (n/n)
Albendazole	65% (56)	35% (53)	67% (64)
Levamisole	92% (56)	49% (53)	69% (64)
Abamectin	26% (56)	60% (53)	89% (64)
Moxidectin	67% (56)	75% (53)	92% (64)
Benzimidazole/levamisole/abamectin combination	99% (27)	80% (36)	91% (40)
Derquantel–abamectin combination	97% (56)	97% (53)	99% (64)
Monepantel	98% (55)	97% (53)	97% (64)
Naphthalophos	94% (18)	*	72% (11)
Closantel	81% (25)	**	**

^a^ Any of the nematodes *H. contortus, T. circumcincta* or *Trichostrongylus* species (less than 95% reduction).

* Too few tests for useful figures.

** Closantel has no significant activity against species other than *H. contortu*s.

Resistance to the ‘triple combination’ of a benzimidazole, levamisole and abamectin varied between genera, presumably according to the performance of the individual anthelmintics, with resistance indicated in only 7% of tests in *H. contortus*, where levamisole remained >95% effective in the majority of tests. Similarly, resistance to the ‘triple combination’ was indicated in only 28% of tests to *Trichostrongylus*, for which abamectin remained relatively effective, but was common in *T. circumcincta* (67% of tests) for which none of the component anthelmintics gave a mean reduction of more than 60%, and for which the combined actives gave a mean reduction of only 80%.

For the newer anthelmintics monepantel and derquantel–abamectin, resistance by individual genera was far less common but with a marked between‐species variation: for *T. circumcincta*, in 21% and 15% of tests for derquantel–abamectin and monepantel, respectively, less prevalent in *H. contortus* (13% for both) and uncommon in *Trichostrongylus* (a prevalence of 3% and 4%, respectively). However, the mean efficacy across all tests remained over 95% for both anthelmintics for all three genera (reductions of 97%–99%).

A smaller number of tests were conducted for the narrow‐spectrum anthelmintic closantel and the ‘mid‐spectrum’ naphthalophos. For closantel, counts of *H. contortus* (for which activity is largely specific) were less than 95% in 15 of 25 tests (60%, with a mean reduction of 81%), although there was a substantial geographic variation in effectiveness, with closantel more effective in winter rainfall regions, where it is used less commonly due to the relatively sporadic occurrence of haemonchosis. Resistance to naphthalophos was indicated in 33% of tests for *H. contortus*, against which it is most commonly used, with a mean reduction of 94%, but reductions were substantially lower for the other genera, for which naphthalophos is considered only partially effective and is usually used in combination with other actives.

## Discussion

Although not conducted as a random survey, the relatively large number of recent FECRT results provides an indication of the present prevalence and severity of drench resistance in sheep worms and a basis for comparisons with previous compilations of test results. The properties involved can be considered as indicative of the wider sheep industry, as they represent a range of locations in the major sheep grazing areas in Australia and were chosen on the basis of relatively large flocks and an interest by the owners in worm control.

The present FECR figures for all species (Table 1) are consistent with previous results indicating resistance to the older anthelmintics to be present on almost all Australian sheep properties and that it has continued to increase. Previous reports show that by the early 1990s, resistance was well‐established in the major nematode genera (*H. contortus*, *T. circumcincta* and *Trichostrongylus* species) to the benzimidazoles and levamisole^16^ and had further increased by the late 1990s.^5^ Resistance to the ML anthelmintics was detected in *H. contortus* ^17^ and *T. circumcincta* ^18^ a few years after the introduction of ivermectin to Australia, with more recent Australia‐wide results^6^ indicating the prevalence for the major species (combined) as 87% for ivermectin, 77% for abamectin and 54% for moxidectin (a more potent and persistent ML^19^, ^20^).

Inferences of changes in resistance figures over time from comparisons of results are qualified by the limitation that the tests were not necessarily conducted on the same properties, but the higher prevalences indicated in the present result collation (for all major genera) of 91% for abamectin and 84% for moxidectin (ivermectin was not included) suggest a further increase in ML resistance. Given the widespread use of the MLs as single‐actives, as well as their use in most combination anthelmintic products and as a long‐acting formulation of moxidectin, the near‐universal failure of this class to provide effective worm control is a major concern. The significant increase in reduced efficacy of the ‘triple combination’ of abamectin plus levamisole and a benzimidazole, from a prevalence of 28% in the 2014 report^6^ to the present 66%, is a further concern.

The test results also confirm that resistance to the newer anthelmintics, monepantel (available in Australia since 2010) and derquantel–abamectin (available from 2014) is no longer a rare occurrence. No cases of resistance were reported for these products by previous reports,^6^, ^7^ but in the present compilation, FECRT reductions (for any species) of less than 95% were indicated in 23% of tests for both monepantel and derquantel–abamectin. The lower prevalence than for other anthelmintic groups presumably reflects the shorter period of exposure of parasites to these more recently introduced actives and possibly the adoption of more sustainable strategies for worm control by some sheep owners at the time of their introduction.

The results for individual nematode genera or species confirm the trends evident from the overall figures, and the substantial differences in efficacy between the genera (Table 2) highlight the importance of identifying the species present. Resistance was especially severe to abamectin in *H. contortus*, with a prevalence of 95% and a mean FECR of only 26%, and of particular concern, abamectin gave a zero egg count reduction in nearly half of the 56 tests. Although the reductions for *H. contortus* were greater for the more potent moxidectin, resistance was nonetheless present in 79% of tests, with a mean FECR of only 67%. Resistance to the MLs was also prevalent in *T. circumcincta* (83% and 70% of tests for abamectin and moxidectin, respectively), with mean reductions of only 60% and 75%. However, ML resistance was far less common for *Trichostrongylus*, with a prevalence of only 30% for both abamectin and moxidectin, and a mean reduction of close to 90% for both, consistent with previous contentions that ML resistance is of greatest concern for *H. contortus* ^21^ and *T. circumcincta*. A similar marked difference between species in efficacy for specific anthelmintics was evident for levamisole against *H. contortus*, with resistance indicated in only 21% of tests and a mean reduction of 92%, in contrast to the comparative severity of levamisole resistance by *T. circumcincta* and *Trichostrongylus*. However, there is little scope to consider anthelmintics effective against only some species for narrow‐spectrum treatments because sheep are usually co‐infected with several worm genera.

It is likely that the considerable between‐genus differences in efficacy explain the better performance of multi‐combination products against species for which at least one component anthelmintic was generally effective (e.g. for *H. contortus*, levamisole, and for *Trichostrongylus*, MLs), in contrast to *T. circumcincta*, for which resistance to all of the older anthelmintics was common and often severe. A key reason for using anthelmintics in combination is to reduce the selection pressure on the individual actives,^22^, ^23^ but in many instances resistance has already developed to all components. While the efficacy of a combination against a particular nematode population at a particular time may exceed 95%, this may not apply with seasonal changes in the proportion of different species, and efficacy should be confirmed before strategic treatments are given.

Narrow‐spectrum anthelmintics offer the possibility of reducing exposure to the broad‐spectrum groups and have a particular role against *H. contortus*. However, for closantel the FECR results were less than 95% effective on 60% of properties, confirming the high prevalence of resistance indicated in results from 2009 to 2012^6^ and earlier. The ‘mid‐spectrum’ anthelmintic naphthalophos is also mainly used in Australia for control of *H. contortus*, although as it has lower and variable efficacy against susceptible strains of the ‘scour worms’ (e.g. *T. circumcincta* and *Trichostrongylus* species), it has been used in combination with other anthelmintics against mixed‐species worm populations (WormBoss website: www.wormboss.com.au). The reduction of less than 95% indicated for naphthalophos in 31% of the current series of tests for *H. contortus* was higher than the 15% reported in the earlier report^6^ but suggests declining efficacy. For both closantel and naphthalophos, confirmation of effectiveness against a particular *H. contortus* population would be necessary before either could be considered an appropriate choice, including when used in combination with other anthelmintics, as resistance to four‐active combination products containing a benzimidazole, levamisole, abamectin and closantel has been reported in *H. contortus* in Australia.^24^ Testing is especially necessary for combination products that contain only abamectin and closantel, as even if effective against *H. contortu*s, other species present would be exposed to abamectin, risking the exacerbation of ML resistance. A further risk of using closantel without confirming efficacy is the reduction in persistent activity against *H. contortus* following the development of resistance, leaving sheep vulnerable to reinfection after a shorter interval than expected.

From the present results it is evident there is no longer a role for the individual‐active use of benzimidazoles, levamisole or the MLs on Australian sheep properties, and that moxidectin as a long‐acting formulation should be used only with monitoring for effectiveness and usually with other anthelmintics (especially as ‘primer’ and ‘exit’ drenches). Similarly, combinations of an ML, benzimidazole and levamisole, and the narrow‐ or mid‐spectrum anthelmintics closantel and naphthalophos for *H. contortus* control can no longer be assumed to be adequately effective unless confirmed by an anthelmintic test. While resistance to the newer anthelmintics, monepantel and the derquantel–abamectin combination is not as advanced in comparison to the older anthelmintics, the demonstration of resistance on some properties and involving all three major worm genera^9^, ^10^, ^11^ indicates that the prevalence is likely to increase unless effective sustainable control measures are adopted. The newer actives would also be most sustainably used in combination with other anthelmintic groups, provided that at least one active has full efficacy. (Monepantel is now available in Australia for use against sheep nematodes only in combination with abamectin.)

Drench resistance management strategies have been recommended in Australia for many years,^25^ especially the adoption of integrated parasite management (IPM) programs that reduce the reliance on anthelmintics for worm control.^26^, ^27^, ^28^ Encouragingly, a series of parasite control surveys of Australian sheep farmers over the last two decades suggest an increasing adoption of the key elements of IPM recommendations.^29^, ^30^ Although there appeared to be no substantial decrease in the average number of anthelmintic treatments, the survey responses indicated an increased use of low worm‐infectivity pastures, appropriate nutritional strategies, the introduction of rams selected for worm resistance and the monitoring of faecal worm egg counts.^30^ Of note, programs intended to provide ‘refugia’ for non‐resistant worm populations^31^, ^32^ had gained some adoption,^30^ despite the acknowledged slow rate of acceptance of novel and relatively complex strategies.^33^

Despite the obvious risk to sheep worm control of using anthelmintics that are only partially effective, relatively few farmers in Australia presently conduct routine drench tests, with testing reported from a mean of just 6% of properties each year in recent years.^30^ Periodic testing for anthelmintic resistance has been strongly advocated for many years, and test methodologies are described in detail on the WormBoss website, but many farmers consider the FECRT procedure (‘DrenchTest’) to be difficult and time‐consuming.^33^ A simple comparison of worm egg counts before and after anthelmintic treatment (‘DrenchCheck’) can provide a useful indication of the likely presence of resistance and its relative severity, although an undifferentiated reduction result may mask underlying resistance to species present in small proportions, and can underestimate the severity of resistance. The increasing availability of rapid and sensitive methods for worm egg counting and highly discriminatory molecular tests for species identification offers the prospect of improved speed and sensitivity of resistance detection,^34^ but the new technologies will need to be generally accepted and commercially attractive to diagnostic laboratories.

Almost certainly, the continued use of anthelmintics to which resistance has developed but is not visually evident is commonly causing sub‐optimal worm control, and resistance will continue to develop with their continued use. The indication that at least a proportion of Australian sheep farmers have adopted more sustainable worm control strategies is timely in view of the narrowing number of effective drench options, but routine drench testing is essential to ensure the appropriate selection of anthelmintics.

## Conclusions

A series of recent drench tests across Australia indicate that resistance by sheep nematodes to the benzimidazole, levamisole and ML anthelmintics has continued to increase, and resistance to a combination of these groups is now present on most properties. Resistance to the newer anthelmintics monepantel and a derquantel–abamectin combination is no longer uncommon, and the effectiveness of the narrow‐spectrum anthelmintics closantel and naphthalophos is variable. Routine testing for anthelmintic efficacy, differentiated to nematode species, is essential to ensure optimal sheep worm control.

## Conflicts of interest and sources of funding

The authors declare no conflicts of interest for the work presented here. NM Rolls is a paid employee of Elanco Australasia and assisted with the preparation of the manuscript. RB Besier is an independent scientist, funded by Elanco Australasia to analyse the data and prepare the publication.

## Data Availability

Data is provided in the text and tables, but the identity of the properties from which the information was derived is confidential. However, the property identity is of no relevance to the compilation of test results.

## References

[1] Shephard R , Webb Ware J , Blomfield B et al. Priority list of endemic diseases for the red meat industry – 2022 update. Project B.AHE.0327. North Sydney, Meat & Livestock Australia Limited, 2022.

[2] Wolstenholme AJ , Fairweather I , Prichard R et al. Drug resistance in veterinary helminths. Trends Parasitol 2004;20:469–476. 10.1016/j.pt.2004.07.010 15363440

[3] Kaplan RM , Vidyashankar AN . An inconvenient truth: global worming and anthelmintic resistance. Vet Parasitol 2012;186:70–78. 10.1016/j.vetpar.2011.11.048 22154968

[4] Coles GC , Bauer C , Borgesteede FHM et al. World Association for the Advancement of Veterinary Parasitology (W.A.A.V.P.) – methods for the detection of anthelmintic resistance in nematodes of veterinary importance. Vet Parasitol 1992;44:35–44. 10.1016/0304-4017(92)90141-u 1441190

[5] Besier RB , Love SCJ . Anthelmintic resistance in sheep nematodes in Australia: the need for new approaches. Aust J Exp Agric 2003;43:1383–1391.

[6] Playford MC , Smith AN , Love S et al. Prevalence and severity of anthelmintic resistance in ovine gastrointestinal nematodes in Australia (2009–2012). Aust Vet J 2014;92:464–471. 10.1111/avj.12271 25424758

[7] Preston S , Piedrafita D , Sandeman M et al. The current status of anthelmintic resistance in a temperate region of Australia; implications for small ruminant farm management. Vet Parasitol 2019;17:100313. 10.1016/j.vprsr.2019.100313 31303219

[8] Constantinoiu C , De Cat S . Lack of efficacy of monepantel against Haemonchus contortus and Trichostrongylus spp in small ruminants. Brisbane, Proceedings of the 4th Australian Veterinary Association (AVA)/New Zealand Veterinary Association (NZVA) Pan Pacific Veterinary Conference, 2015.

[9] Sales N , Love SCJ . Resistance of *Haemonchus* sp. to monepantel and reduced efficacy of a derquantel/abamectin combination confirmed in sheep in NSW, Australia. Vet Parasitol 2016;228:193–196. 10.1016/j.vetpar.2016.08.016 27692326

[10] George SD , George AJ , Stein PA et al. The comparative efficacy of abamectin, monepantel and an abamectin/derquantel combination against fourth‐stage larvae of a macrocyclic lactone‐resistant *Teladorsagia* spp. isolate infecting sheep. Vet Parasitol 2012;188:190–193. 10.1016/j.vetpar.2012.03.001 22459111

[11] Lamb J , Elliott T , Chambers M et al. Broad spectrum anthelmintic resistance of *Haemonchus contortus* in northern NSW of Australia. Vet Parasitol 2017;241:48–51. 10.1016/j.vetpar.2017.05.008 28579030

[12] Wood IB , Amaral NK , Bairden K et al. World Association for the Advancement of Veterinary Parasitology (WAAVP) second edition of guidelines for evaluating the efficacy of anthelmintics in ruminants (bovine, ovine, caprine). Vet Parasitol 1995;58:181–213. 10.1016/0304-4017(95)00806-2 7571325

[13] Hutchinson GW . Nematode parasites of small ruminants, camelids and cattle diagnosis with emphasis on anthelmintic efficacy and resistance testing. Camden, Australia and New Zealand Standard Diagnostic Procedures, 2009. Available at: https://www.agriculture.gov.au/agriculture-land/animal/health/laboratories/procedures/anzsdp/nematode-parasites-ruminants.

[14] van Wyk JA , Cabaret J , Michael LM . Morphological identification of nematode larvae of small ruminants and cattle simplified. Vet Parasitol 2004;119:277–306. 10.1016/j.vetpar.2003.11.012 15154594

[15] Dikmans G , Andrews JS . A comparative morphological study of the infective larvae of the common nematodes parasitic in the alimentary tract of sheep. Trans Am Microsc Soc 1933;52:1–25.

[16] Overend DJ , Phillips ML , Poulton AL et al. Anthelmintic resistance in Australian sheep nematode populations. Aust Vet J 1994;71:117–121. 10.1111/j.1751-0813.1994.tb03352.x 8048907

[17] Le Jambre LF . Ivermectin‐resistant *Haemonchus contortus* in Australia. Aust Vet J 1993;70:357. 10.1111/j.1751-0813.1993.tb00887.x 8240179

[18] Swan N , Gardner J , Besier RB et al. A field case of ivermectin resistance in sheep in Western Australia. Aust Vet J 1994;71:302–303. 10.1111/j.1751-0813.1994.tb03452.x 7818442

[19] Le Jambre LF , Dobson RJ , Lenane IJ et al. Selection for anthelmintic resistance by macrocyclic lactones in *Haemonchus contortus* . Int J Parasitol 1999;29:1101–1111. 10.1016/s0020-7519(99)00074-0 10501620

[20] Besier RB , Kahn LP , Sargison ND et al. The diagnosis, treatment and management of *Haemonchus contortus* in small ruminants. Adv Parasitol 2016;93:181–238. 10.1016/bs.apar.2016.02.024 27238006

[21] Kotze AC , Prichard RK . Anthelmintic resistance in *Haemonchus contortus*: history, mechanisms and diagnosis. Adv Parasitol 2016;93:397–428. 10.1016/bs.apar.2016.02.012 27238009

[22] Leathwick DM , Hosking BC , Bisset SA et al. Managing anthelmintic resistance: is it feasible in New Zealand to delay the emergence of resistance to a new anthelmintic class? NZ Vet J 2009;57:181–192. 10.1080/00480169.2009.36900 19649011

[23] Bartram DJ , Leathwick DM , Taylor MA et al. The role of combination anthelmintic formulations in the sustainable control of sheep nematodes. Vet Parasitol 2012;186:151–158. 10.1016/j.vetpar.2011.11.030 22245073

[24] Baker E , George SD , Stein PA et al. Efficacy of monepantel and anthelmintic combinations against multiple‐resistant *Haemonchus contortus* in sheep, including characterisation of the nematode isolate. Vet Parasitol 2012;186:513–517. 10.1016/j.vetpar.2011.11.060 22169404

[25] Leathwick DM , Besier RB . The management of anthelmintic resistance in grazing ruminants in Australasia – strategies and experiences. Vet Parasitol 2014;204:44–54. 10.1016/j.vetpar.2013.12.022 24439840

[26] Waller PJ . International approaches to the concept of integrated control of nematode parasites of livestock. Int J Parasitol 1999;29:155–164. 10.1016/s0020-7519(98)00178-7 10048828

[27] Kahn LP , Woodgate RG . Integrated parasite management: products for adoption by the Australian sheep industry. Vet Parasitol 2012;186:58–64. 10.1016/j.vetpar.2011.11.046 22154258

[28] Kotze AC , Hunt PW . The current status and outlook for insecticide, acaricide and anthelmintic resistances across the Australian ruminant livestock industries: assessing the threat these resistances pose to the livestock sector. Aust Vet J 2023;101:321–333. 10.1111/avj.13267 37401786

[29] Colvin AF , Reeve I , Peachey B et al. Benchmarking Australian sheep parasite control practices: a national online survey. Anim Prod Sci 2020;61:237–245.

[30] Colvin AF , Reeve I , Thompson LJ et al. Benchmarking Australian sheep parasite control: changes in gastrointestinal nematode control practices reported from surveys between 2003 and 2019. Vet Parasitol Reg Stud Rep 2021;26:100653. 10.1016/j.vprsr.2021.100653 34879963

[31] Prichard RK , Hall CA , Kelly JD et al. The problem of anthelmintic resistance in nematodes. Aust Vet J 1980;56:239–251. 10.1111/j.1751-0813.1980.tb15983.x 7002142

[32] Hodgkinson JE , Kaplan RM , Kenyon F et al. Refugia and anthelmintic resistance: concepts and challenges. Int J Parasitol Drugs Drug Resist 2019;10:5–57. 10.1016/j.ijpddr.2019.05.001 PMC6531808 31125837

[33] Woodgate RG , Love S . WormKill to WormBoss – can we sell sustainable sheep worm control? Vet Parasitol 2012;186:51–57. 10.1016/j.vetpar.2011.11.045 22153257

[34] Playford M , Besier R . Gastrointestinal nematode parasites of grazing ruminants: a comprehensive literature review of diagnostic methods for quantifying parasitism, larval differentiation and measuring anthelmintic resistance. NZ Vet J 2024;73:1–16. 10.1080/00480169.2024.2415029. 39522537

